# Metabolic factors influencing the efficacy of recombinant human growth hormone therapy in children with short stature

**DOI:** 10.3389/fendo.2025.1691509

**Published:** 2025-11-06

**Authors:** Xueyu Zhong, Yang Chen, Geng Liu, Zhenhai Cui, Kuanhong Luo, Zhixin Wu, Kangli Xiao, Huiqing Li

**Affiliations:** 1 Department of Endocrinology, Union Hospital, Tongji Medical College, Huazhong University of Science and Technology, Wuhan, China; 2 Diabetes and Metabolic Disease Clinical Research Center of Hubei Province, Wuhan, China

**Keywords:** recombinant human growth hormone, short stature, children, insulin resistance, hyperinsulinemia

## Abstract

**Objective:**

This study analyzed metabolic indicators and height gain in short-statured children within the first year of recombinant human growth hormone (rhGH) therapy, identifying predictive factors for treatment efficacy.

**Methods:**

A retrospective analysis of 72 children with short stature (growth hormone deficiency or idiopathic short stature) receiving rhGH therapy (January 2022 to January 2024) was performed. Data included height, weight, age, skeletal age (SA), and laboratory results (IGF1, fasting glucose, insulin, C-peptide, thyroid function, lipids). Analyses focused on height standard deviation score (HSDS), HSDS for SA, and factors associated with 12-month changes in HSDS for SA (△HSDS for SA).

**Results:**

The mean initial rhGH dose was 0.053 ± 0.010mg/kg/day, with a mean starting age of 8.36 ± 2.24 years. Significant increases in HSDS and HSDS for SA were observed after 12 months. △HSDS for SA negatively correlated with baseline homeostasis model assessment of insulin resistance (HOMA-IR) and fasting insulin, and positively correlated with baseline free triiodothyronine (FT3). Children with △HSDS for SA>0.5 had lower baseline insulin and HOMA-IR, and higher FT3, high-density lipoprotein cholesterol (HDL), and hemoglobin.

**Conclusions:**

Insulin resistance, hyperinsulinemia, FT3, and HDL determine rhGH efficacy in short-statured children. Metabolic profiling optimizes rhGH therapy, and targeting insulin resistance may improve growth outcomes.

## Introduction

Short stature is a complex clinical phenomenon, typically defined as a height below -2 standard deviations (SD) or below the 3rd percentile compared to individuals of the same age and sex, encompassing conditions such as growth hormone deficiency (GHD) and idiopathic short stature (ISS) (1). Recombinant human growth hormone (rhGH) is a cornerstone for managing short stature in children with GHD and ISS: its safety and efficacy have been established, and it is widely used in clinical practice (2, 3). However, individual variability in the growth response to rhGH remains a major clinical challenge. Most studies exploring factors influencing rhGH efficacy have focused on baseline height, age, skeletal age (SA), body mass index (BMI), insulin-like growth factor-1 (IGF1), growth hormone stimulation test responses, and initial rhGH dosage (4–10). In contrast, the impact of basal metabolic status on early height growth during rhGH therapy has been less explored.

Growth hormone (GH) influences glucose metabolism by enhancing hepatic gluconeogenesis and glycogenolysis, while reducing peripheral glucose utilization (11). Hyperinsulinemia disrupts the physiological balance between insulin and growth hormone (12): it can induce elevated IGF1 secretion (which subsequently suppresses GH to subnormal levels) and directly inhibit GH synthesis and release, leading to a negative correlation between insulin and GH levels (13). Insulin resistance is closely associated with GH-mediated metabolic regulation. This suggests basal metabolic status may modulate GH’s growth-promoting effects, but its specific role in determining rhGH treatment response remains poorly characterized. Current research has focused on insulin and the homeostasis model assessment of insulin resistance (HOMA-IR) levels after rhGH therapy, rather than exploring the predictive value of baseline insulin and HOMA-IR in children with short stature for rhGH response.

This study aims to explore the predictive role of baseline insulin and HOMA-IR levels in treatment response through a retrospective analysis of data from children with short stature who received rhGH therapy between 2022 and 2024, providing theoretical support for personalized clinical treatment.

## Materials and methods

This retrospective study enrolled 72 children diagnosed with GHD or ISS at Department of Endocrinology, Union Hospital, Tongji Medical College, Huazhong University of Science and Technology between January 2022 and January 2024. Children who visited Union Hospital underwent further examinations if they met any of the following criteria (14): 1) height below -2 SD or the 3rd percentile of the normal growth curve for Chinese children of the same chronological age (CA) and sex (15); 2) target height (TH) standard deviation score (SDS) < 1.6; 3) height standard deviation score (HSDS) decreased significantly (> 1.0 SD/year); or 4) growth velocity ≤5 cm/year. GHD was diagnosed if the serum GH peak was <10 μg/L in two different GH stimulation tests (using insulin, L-dopa, or arginine as stimulants). ISS was diagnosed if no evidence of systemic, endocrine, nutritional, chromosomal abnormalities, or genetic variations was identified in the patients. A total of 205 children were diagnosed with either GHD or ISS, among whom 72 received rhGH treatment. All children diagnosed with GHD or ISS who initiated rhGH therapy at the Department of Endocrinology, Union Hospital, between January 2022 and January 2024 were included in this study. Exclusions were limited to children who did not initiate rhGH therapy, with documented reasons including financial constraints (42%), concerns about adverse effects (32%), parental refusal of injectable treatment (16%), and lack of understanding of treatment necessity (10%). The study protocol was approved by the Ethics Committee of Union Hospital, Tongji Medical College, Huazhong University of Science and Technology (approval number (2025):0420). Informed consent was not required.

In this study, we selected height changes after 12 months of rhGH treatment to analyze therapeutic efficacy. The following indicators and parameters at baseline and at 12 months after rhGH initiation were evaluated: height, weight, body mass index (BMI), HSDS, skeletal age (SA), height standard deviation score for skeletal age (HSDS for SA), IGF1, IGF1 SDS, fasting blood glucose, fasting insulin, HOMA-IR, HOMA-β, fasting C-peptide, free triiodothyronine (FT3), free thyroxine (FT4), thyroid-stimulating hormone (TSH), total cholesterol (TC), low-density lipoprotein cholesterol (LDL), high-density lipoprotein cholesterol (HDL), triglycerides (TG), complete blood count, total bilirubin, alanine transaminase (ALT), aspartate transaminase (AST), and alkaline phosphatase. Clinical factors associated with the 12-month change in HSDS for SA (△HSDS for SA) were analyzed. Unless otherwise specified, results are expressed as mean ± standard deviation SD.

### Statistical analysis

The normality of data distribution was tested using the Shapiro-Wilk test. Normally distributed data were presented as mean ± SD. Non-normally distributed data were described using median and interquartile range (IQR). For continuous variables, two-sample t-tests and the Mann-Whitney U test were used for normally and non-normally distributed data, respectively. Pearson correlation analysis was performed to assess correlations between variables. Linear regression analysis was used to evaluate the association between the variables of interest and △HSDS for SA. Multiple linear regression analysis with a backward elimination method was used to adjust for the effects of potential confounding factors. Statistical analyses were performed using IBM SPSS Statistics 27.0 and GraphPad Prism 7.0 software, with a P value <0.05 considered statistically significant.

## Results

A total of 72 children (37 males and 35 females) were included in the study. The starting mean age of the children was 8.36 ± 2.24 years, with a mean HSDS of -1.78 ± 0.74. Skeletal age/chronological age ratio (SA/CA) was 0.86 ± 0.14, indicating delayed SA. The dose of GH injection for all researchers was 0.053 ± 0.010mg/kg/day, and ISS Group was higher than GHD group (0.057 ± 0.010 vs. 0.047 ± 0.010, *p* < 0.05). Detailed demographic and baseline characteristics are presented in **Table 1**.

**Table 1:** Baseline demographics by diagnostic category.

Characteristic	ALL		GHD		ISS	
	n (%)	Mean ± SD	n (%)	Mean ± SD	n (%)	Mean ± SD
Gender						
Male	37(51.39)	–	21(29.17)	–	16(22.22)	–
Female	35(48.61)	–	16(22.22)	–	19(26.39)	–
Age (y)	72	8.36 ± 2.24	37	8.49 ± 2.01	35	8.21 ± 2.48
BMI (kg/m^2^)	72	16.52 ± 2.47	37	16.74 ± 2.42	35	16.30 ± 2.53
HSDS	71	-1.78 ± 0.74	37	-1.83 ± 0.60	34	-1.73 ± 0.87
HSDS for SA	72	-0.63 ± 0.82	37	-0.46 ± 0.87	35	-0.80 ± 0.74
IGF1 SDS	50	-1.19 ± 0.87	28	-1.13 ± 0.93	22	-1.26 ± 0.80
GH dose (mg/kg/day)	69	0.053 ± 0.010	35	0.047 ± 0.010	34	0.057 ± 0.010*

BMI, body mass index; GH, growth hormone; GHD, growth hormone deficiency; HSDS, height standard deviation score; IGF1, insulin-like growth factor 1; ISS, idiopathic short stature; SD, standard deviation; SDS, standard deviation score. *p < 0.05 vs. GHD.

**Figure 1:**
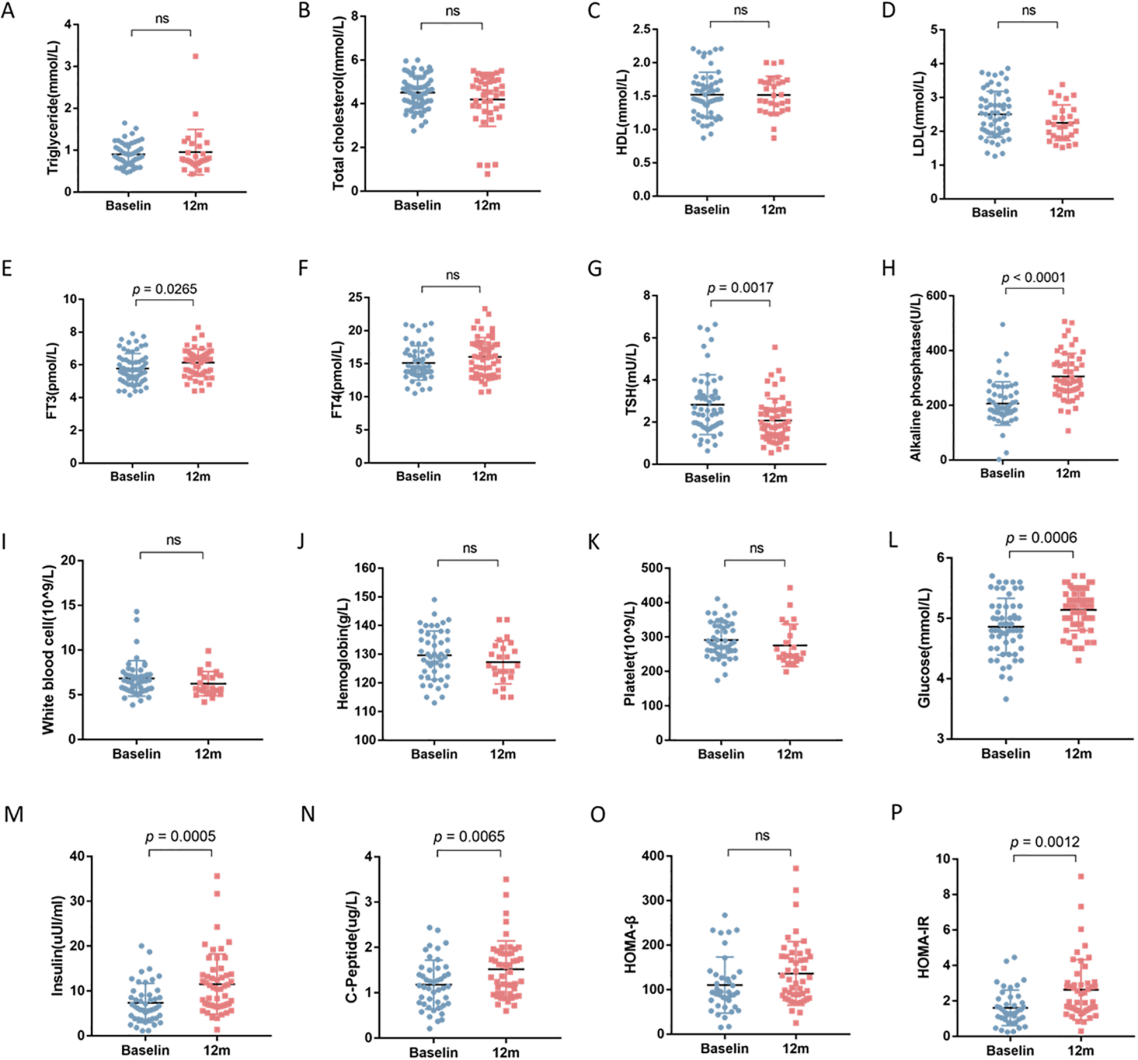
The impact of 12-month rhGH therapy on lipid profile, thyroid function, complete blood count and glucose homeostasis. ns, not significant. **(A)** Triglyceride (TG). **(B)** Total cholesterol (TC). **(C)** High density lipoprotein (HDL). **(D)** Low density lipoprotein (LDL). **(E)** Free triiodothyronine (FT3). **(F)** Free thyroxine (FT4). **(G)** Thyroid stimulating hormone (TSH). **(H)** Alkaline phosphatase (ALP). **(I)** White blood cell count (WBC). **(J)** Hemoglobin (HGB). **(K)** Platelet (PLT). **(L)** Fasting blood glucose (FBG). **(M)** Fasting insulin (FINS). **(N)** C-peptide (CP). **(O)** Homeostasis model assessment of β cell function (HOMA-β). **(P)** Homeostatic model assessment of insulin resistance (HOMA-IR).

After 12 months of rhGH therapy, the mean height increment from baseline was 10.13± 2.07cm, with a significant improvement in HSDS by 0.73 ± 0.31. The △HSDS for SA was 0.46 ± 0.53, confirming the effectiveness of rhGH therapy (**Table 2**). At 12 months, significant increases were observed in fasting blood glucose, fasting insulin, fasting C-peptide, HOMA-IR, and alkaline phosphatase levels compared to baseline (all *P* < 0.05). Lipid profiles and complete blood count did not show significant variations during treatment. Additionally, FT3 levels increased, TSH levels decreased, while FT4 remained unchanged (**Figure 1**).

**Table 2:** Changes in anthropometric measurements throughout 12 months of rhGH therapy.

Characteristic	ALL		GHD		ISS	
	n	(Mean ± SD)	n	(Mean ± SD)	n	(Mean ± SD)
BMI (kg/m^2^)	59	16.78 ± 2.40	33	16.75 ± 2.57	26	16.82 ± 2.20
HSDS	72	-1.08 ± 0.76	37	-1.05 ± 0.54	35	-1.10 ± 0.94
△HSDS	71	0.73 ± 0.32	37	0.77 ± 0.30	34	0.69 ± 0.33
HSDS for SA	72	-0.17 ± 0.77	37	-0.04 ± 0.84	35	-0.31 ± 0.66
△HSDS for SA	72	0.46 ± 0.53	37	0.43 ± 0.54	35	0.49 ± 0.52
IGF1 SDS	50	0.35 ± 1.39	27	0.38 ± 1.56	23	0.30 ± 1.18
△IGF1 SDS	38	1.27 ± 1.23	22	1.33 ± 1.34	16	1.20 ± 1.11

BMI, body mass index; GH, growth hormone; GHD, growth hormone deficiency; HSDS, height standard deviation score; IGF1, insulin-like growth factor 1; ISS, idiopathic short stature; SD, standard deviation; SDS, standard deviation score.

**Figure 2:**
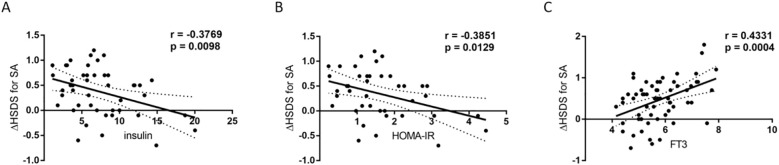
Correlations between baseline insulin, HOMA-IR, FT3 and △HSDS for SA after 12 months of rhGH therapy. **(A)** Relationship between baseline insulin levels and △HSDS for SA after 12 months of rhGH therapy. The total correlation plus the 95% confidence interval is shown (r=-0.3769; *p* = 0.0098; Spearman coefficient of correlation). **(B)** Relationship between baseline HOMA-IR and △HSDS for SA after 12 months of rhGH therapy. The total correlation plus the 95% confidence interval is shown (r=-0.3851; *p* = 0.0129; Spearman coefficient of correlation). **(C)** Relationship between baseline FT3 levels and △HSDS for SA after 12 months of rhGH therapy. The total correlation plus the 95% confidence interval is shown (r=0.4331; *p* = 0.0004; Spearman coefficient of correlation).

Correlation analysis revealed that baseline fasting insulin (r = -0.3769, *p* = 0.0098) and baseline HOMA-IR (r = -0.3851, *p* = 0.0129) were negatively correlated with ΔHSDS for SA after 12 months of rhGH therapy, and the correlations were statistically significant. In contrast, baseline FT3 (r = 0.4331, *p* = 0.0004) was positively correlated with ΔHSDS for SA, and the correlation was significant (**Figure 2**). These results suggest that baseline insulin, HOMA-IR, and FT3 may play a role in influencing ΔHSDS for SA.

In univariable linear regression analysis, we found significant positive association between △HSDS for SA and baseline FT3 (β=0.242, *p* < 0.001). There was significant negative association between △HSDS for SA and baseline insulin (β=-0.041, *p* = 0.010), and baseline HOMA-IR (β=-0.203, *p* = 0.005). In the adjusted model, △HSDS for SA was positively correlated with hemoglobin (β=0.021, *p* = 0.025) and negatively correlated with HOMA-IR (β=-0.179, *p* = 0.029). Consistently, reverse linear regression showed significantly positively association between △HSDS for SA and hemoglobin (β=0.019, *p* = 0.040), negatively association between △HSDS for SA and HOMA-IR (β=-0.190, *p* = 0.013) (**Table 3**). Overall, baseline insulin, HOMA-IR, FT3, and hemoglobin likely play key roles in the outcomes. While other variables showed weak current associations, they may be relevant under different research conditions.

**Table 3:** Multiple linear regression analysis assessed association between study baseline variables and △HSDS for SA.

Baseline variables	Univariable linear regression Beta(95%CI)	*P* value	Adjusted model^a^ Beta(95%CI)	*P* value	Backward linear regression Beta (95%CI)	*P* value
Age	0.04(-0.01, 0.10)	0.113	–	–	–	–
IGF1 SDS	-0.10(-0.25, 0.05)	0.183	–	–	–	–
GH dose	0.037(-0.044, 0.117)	0.366	–	–	–	–
glucose	-0.159(-0.423, 0.105)	0.232	–	–	–	–
insulin	-0.041(-0.071,-0.010)	***0.010***	–	–	–	–
C-peptide	0.006(-0.163, 0.175)	0.939	–	–	–	–
HOMA-IR	-0.203(-0.343, -0.064)	***0.005***	-0.179(-0.338, -0.020)	***0.029***	-0.190(-0.338, -0.043)	***0.013***
HOMA-β	0.000(-0.001, 0.001)	0.766	–	–	–	–
FT3	0.242(0.113, 0.371)	***<0.001***	0.143(-0.051, 0.336)	0.140	0.171(-0.014, 0.355)	0.068
FT4	0.023(-0.024, 0.070)	0.330	–	–	–	–
TSH	0.083(-0.005, 0.171)	0.065	0.065(-0.052, 0.181)	0.262	–	–
TG	-0.234(-0.468, 0.000)	0.050	-0.242(-0.505, 0.021)	0.069	-0.218(-0.462, 0.025)	0.077
TC	-0.058(-0.228, 0.112)	0.498	–	–	–	–
HDL	0.345(-0.016, 0.706)	0.061	-0.078(-0.563, 0.407)	0.742	–	–
LDL	-0.106(-0.293, 0.080)	0.257	–	–	–	–
WBC	0.012(-0.057, 0.081)	0.724	–	–	–	–
RBC	0.021(-0.397, 0.439)	0.919	–	–	–	–
Hemoglobin	0.014(-0.002, 0.030)	0.091	0.021(0.003, 0.040)	***0.025***	0.019(0.001, 0.036)	***0.040***
Platelet	0.000(-0.003, 0.002)	0.837	–	–	–	–

a Adjusted linear regression: All variables with *p* value less than 0.1 were included in the model. Bold and italicized values indicate a p value < 0.05.

The subjects were then divided into two groups based on the median △HSDS for SA of 0.5. Univariate analysis showed that there was no significant difference in the dosage of rhGH between two groups. The levels of fasting insulin and HOMA-IR in the △HSDS for SA > 0.5 group were significantly lower, while the levels of FT3, HDL, and hemoglobin were notably higher (**Table 4**). These findings highlight the potential role of metabolic indicators in predicting the efficacy of growth hormone therapy.

**Table 4:** Univariate analysis results of metabolic indicators for △HSDS for SA throughout 12 months of rhGH therapy.

Baseline variables	△HSDS for SA		t/Z	*P*
	≤0.5 (n=40)	>0.5 (n=32)		
GH dose (mg/kg/day)	0.053 ± 0.013	0.053 ± 0.010	-0.466	0.643
IGF1 SDS	-1.01 ± 1.01	-1.42 ± 0.61	1.766	0.084
glucose(mmol/L)	4.91 ± 0.47	4.79 ± 0.47	0.925	0.359
insulin(uU/ml)	8.37 ± 4.82	5.73 ± 3.09	2.259	***0.029***
C-peptide(ug/L)	1.14(0.72, 1.54)	1.25(0.76, 1.56)	-0.323	0.746
HOMA-IR	1.84 ± 1.11	1.16 ± 0.51	2.667	***0.011***
HOMA-β	93.81(61.00, 171.11)	101.89(76.00,135.54)	-0.055	0.956
FT3(pmol/L)	5.52 ± 0.81	6.07 ± 0.96	-2.467	***0.016***
FT4(pmol/L)	15.25 ± 2.58	14.99 ± 2.67	0.371	0.712
TSH(uU/L)	2.34(1.70, 3.12)	3.12(1.92, 3.90)	-1.636	0.102
TG(mmol/L)	0.89(0.71, 1.07)	0.83(0.68, 1.19)	0.000	1.000
TC(mmol/L)	4.48 ± 0.74	4.40 ± 0.74	0.427	0.671
HDL(mmol/L)	1.44 ± 0.32	1.62 ± 0.34	-2.039	***0.046***
LDL(mmol/L)	2.60 ± 0.67	2.38 ± 0.68	1.254	0.215
WBC(10^9/L)	6.78 ± 2.29	6.87 ± 1.55	-0.155	0.877
RBC(10^12/L)	4.61 ± 0.33	4.59 ± 0.33	0.133	0.895
Hemoglobin(g/L)	126.89 ± 7.68	131.76 ± 8.28	-2.108	***0.041***
Platelet(10^9/L)	293.52 ± 55.04	288.90 ± 49.25	0.301	0.764

Bold and italicized values indicate a p value < 0.05.

## Discussion

In this study the mean age at treatment initiation was 8.36 ± 2.24 years. A potential factor is that most parents and primary care physicians in China may lack adequate awareness to identify if short statured children need therapy. Additionally, economic considerations and concerns about rhGH related side effects may contribute to hesitation in pursuing this treatment. Our research reveals that even under standardized rhGH dosage adjustment protocols, significant interpatient differences in height growth response persist. Notably, we identified several key metabolic parameters—including baseline insulin resistance (as measured by HOMA-IR), hyperinsulinemia, FT3, and HDL—as significant predictors of 12-month height gain (ΔHSDS for SA) in rhGH-treated patients. These results highlight the critical role of basal metabolic factors in modulating rhGH treatment efficacy. Incorporating metabolic profiling into routine clinical assessments and tailoring therapeutic interventions based on these metabolic indicators may facilitate enhanced treatment outcomes and optimization of individualized rhGH therapy strategies. Such an approach could contribute to more precise prediction of growth responses and personalized management of children with GHD or ISS.

A central finding of our study is the significant negative correlation between baseline HOMA-IR, fasting insulin levels, and ΔHSDS for SA, with HOMA-IR retaining significance in adjusted and backward linear regression models. This aligns with accumulating evidence that insulin-GH crosstalk plays a pivotal role in growth regulation. Insulin and GH exert reciprocal influences: while GH promotes lipolysis and insulin counteracts this effect, chronic hyperinsulinemia and insulin resistance can impair GH signaling. As demonstrated in preclinical studies, hyperinsulinemia suppresses pulsatile GH secretion by inhibiting somatotroph function and enhancing somatostatin release, thereby reducing GH bioavailability (12). Moreover, insulin resistance may directly blunt GH-induced intracellular signaling, as insulin has been shown to downregulate GH receptor (GHR) expression and impair JAK2/STAT5 activation—key pathways for GH-mediated growth promotion (16).

Previous literatures have reported the impact of rhGH treatment on insulin and HOMA-IR, but have not indicated the influence of baseline insulin and HOMA-IR levels on the efficacy of rhGH therapy. The study by Capalbo et al. showed that after 1 year of GH treatment, the increase in HOMA-IR in children with GHD was significantly higher than that in the control group; however, after 5 years of GH treatment, there was no significant difference in HOMA-IR between children with GHD and the control group at 5 years, suggesting that the degree of insulin resistance after long-term treatment is consistent with the physiological changes in healthy pubertal children (17). A study by Ciresi et al. indicated that after 12 months of GH treatment in children with GHD, HOMA-IR increased from 1.1 ± 1.2 to 2.0 ± 1.4, and was negatively correlated with insulin sensitivity measured by the clamp test (18). In our study, we also observed that HOMA-IR after 12 months of rhGH treatment was higher than the baseline value, which is consistent with the results of the aforementioned studies. Notably, baseline HOMA-IR, a practical surrogate for insulin resistance, emerges as a robust predictor for good response to rhGH in our analysis, underscoring its utility in clinical practice. This suggests that assessing insulin resistance at baseline could help stratify patients likely to benefit from adjunctive therapies targeting insulin sensitivity to enhance rhGH responsiveness, as hyperinsulinemia is increasingly recognized as a key disruptor of the insulin-GH balance (19).

The study of Horikawa et al. in children born small for gestational age (SGA) has shown that rhGH therapy improves lipid profiles (20). After 260 weeks of treatment with a high dose of rhGH (0.067 mg/kg/day), TC and LDL decreased significantly; whereas after 260 weeks of treatment with a low dose of rhGH (0.033 mg/kg/day), HDL increased significantly. Existing studies have shown that GH promotes the hydrolysis of triglycerides into free fatty acids (FFA) by activating hormone-sensitive lipase in adipose tissue, and increases the transport of FFA to muscles and the liver, thereby reducing circulating TG levels. Meanwhile, GH upregulates the expression of LDL receptors in the liver and inhibits proprotein convertase subtilisin/kexin type 9 (PCSK9), which promotes the clearance of LDL and reduces plasma LDL levels (21). In our study, the lipid profile showed no significant changes after 12 months of rhGH treatment. This lack of detectable lipid change may result from two potential factors: First, the 12-month follow-up was insufficient to capture meaningful alterations, 12-month exposure only triggered subtle, statistically insignificant lipid metabolism adjustments. Second, the dosage of rhGH differed from prior studies. But we found that baseline HDL is associated with ΔHSDS for SA, with higher HDL correlating with better growth outcomes. While the mechanisms underlying this association require further exploration, HDL is known to modulate metabolic homeostasis, including insulin sensitivity. Low HDL is a hallmark of atherogenic dyslipidemia, often coexisting with insulin resistance, which may indirectly impair GH action (22). Additionally, HDL possesses anti-inflammatory properties and may promote endothelial function, potentially facilitating nutrient delivery to growing tissues. Our data extend this link by suggesting that baseline HDL could serve as a prognostic marker for rhGH efficacy, possibly by reflecting overall metabolic health.

FT3, a biologically active thyroid hormone, showed a positive correlation with ΔHSDS for SA in our study. Thyroid hormones are well-recognized regulators of linear growth, acting synergistically with GH to stimulate chondrocyte proliferation in the growth plate. FT3 enhances GH sensitivity by upregulating GHR expression and potentiating GH-induced IGF1 production in the liver and peripheral tissues (16). Conversely, hypothyroidism is a known cause of growth retardation, which is reversible with thyroid hormone replacement. Our findings underscore that maintaining optimal thyroid function is pivotal for optimizing rhGH efficacy in children. Clinically, this highlights the necessity of routine thyroid function monitoring (including FT3, FT4, and TSH) during rhGH therapy, as even subtle declines in FT3 could blunt growth responses—emphasizing that thyroid health is a cornerstone of successful rhGH - driven growth.

Our findings confirm a positive relationship between ΔHSDS for SA and hemoglobin levels, with higher hemoglobin concentrations observed in the subgroup showing more favorable ΔHSDS for SA. This association reinforces hemoglobin’s critical role in childhood linear growth. Biologically, hemoglobin sustains tissue oxygenation, a prerequisite for normal growth plate chondrocyte function—impairment of which, due to low hemoglobin, disrupts longitudinal bone growth (23). Consistent with this, studies in children with nonglomerular chronic kidney disease or sickle cell disease have linked reduced hemoglobin to growth impairment (24, 25). Additionally, nutritional interventions elevating hemoglobin improve height outcomes (26). Clinically, hemoglobin status should be considered in evaluating growth potential, particularly during growth hormone therapy.

Our results highlight the need to integrate metabolic assessments into the pre-rhGH treatment workup. For children with elevated HOMA-IR or hyperinsulinemia, targeted interventions to improve insulin sensitivity—such as dietary modifications, physical activity, or metformin—may enhance rhGH-induced growth. Meanwhlie, optimizing thyroid function and addressing low HDL could further improve outcomes. These strategies align with a growing consensus that metabolic health is an integral component of growth regulation, and its importance may be on par with traditional factors such as growth hormone dosage or SA (19).

### Limitations

Several limitations should be acknowledged. Our sample size is relatively small, and the 12-month follow-up period may not capture long-term growth dynamics. Larger, longitudinal studies are needed to validate these findings and explore whether improvements in insulin resistance translate to sustained growth benefits. HOMA-IR, while clinically convenient, is an indirect measure of insulin resistance; more precise methods, such as hyperinsulinemic-euglycemic clamps, could provide deeper insights into the mechanistic link between insulin action and GH responsiveness.

## Conclusion

In conclusion, our study identifies insulin resistance, hyperinsulinemia, FT3, and HDL as key determinants of rhGH efficacy in children with short stature. These findings underscore the importance of metabolic profiling in optimizing rhGH therapy and suggest that targeting insulin resistance could be a promising strategy to enhance growth outcomes, supported by evidence that suppressing hyperinsulinemia restores GH secretion and action.

## Data Availability

The original contributions presented in the study are included in the article/supplementary material. Further inquiries can be directed to the corresponding authors.
